# Current issues in heartworm chemotherapy

**DOI:** 10.1186/s13071-026-07327-y

**Published:** 2026-03-18

**Authors:** Timothy G. Geary

**Affiliations:** 1 McGill University, Montreal, QC Canada; 2 Queen’s University Belfast, Belfast, Northern Ireland UK

**Keywords:** Heartworms, Macrocyclic lactones, Anthelmintic resistance, Canine hookworms, Emodepside

## Abstract

Several issues that affect prevention and treatment of heartworm infections require more intensive research. The incidence of heartworm infection in the USA is increasing, but the factors that underlie this trend remain incompletely understood. The contributions of climate change, vector range expansion, client compliance and resistance to macrocyclic lactones (ML) are likely interrelated and require investigation. Molecular-level research has not yet identified the causative mechanisms underlying ML resistance (MLR), but surveys of genomic markers associated with the trait reveal worrying trends in the presence and frequency of these resistance alleles. Research is needed to confirm the phenotypic relevance of these markers and to identify the gene(s) responsible for it. Developing highly inbred strains of MLR heartworms may be necessary but would require multigenerational studies of targeted breeding of selected parasites in dogs. A second issue of concern for veterinarians is the increasing extra-label use of emodepside products for the treatment of multiple anthelmintic drug resistant (MADR) canine hookworms (*Ancylostoma caninum*), which are already common throughout the USA. Emodepside is not approved for use in dogs in the USA, but a cat topical product containing the drug is, and is being used orally in hookworm-infected dogs. Emodepside has activity against larval and adult stages of many filarial parasites and the safety of this drug in heartworm-infected dogs has not been reported. It is perhaps unlikely that such studies will be undertaken, given the lack of economic motivation. Nonetheless, a review of the relevant literature leads to the conclusion that the status of heartworm infection in a dog bearing an apparently MADR hookworm infection be determined before starting treatment with emodepside, with caution exercised should it ensue.

**Graphical Abstract:**

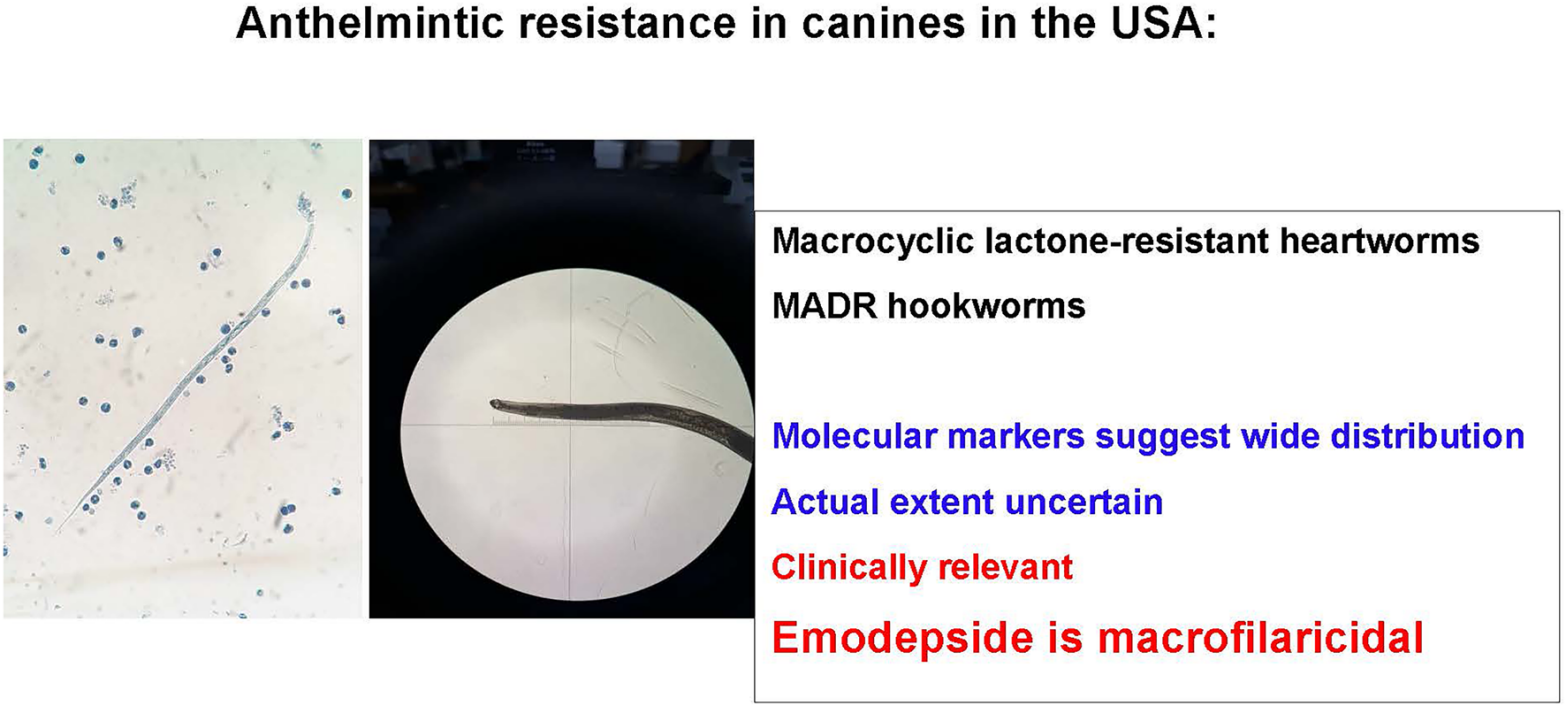

## Background

Resistance to chemotherapeutic agents is increasingly problematic for all infectious diseases, including those caused by parasites. Anthelmintic resistance (AR) has plagued the treatment of veterinary parasites for decades. First described in ruminants, AR is known in virtually all host species that are treated with these drugs. Initial reports of loss-of-efficacy cases in dogs thought to have been treated with macrocyclic lactone (ML) preventatives but found to be infected with the canine heartworm *Dirofilaria immitis* were highlighted in 2005 [1]. Subsequent research revealed that ML-resistant (MLR) isolates differed significantly from susceptible (wild type, WT) populations at the genome level [2] and survived previously 100% effective preventative ML treatment in experimentally infected dogs [3, 4]. The phenotype of MLR in heartworms is clinically evident in the ability of L4 stages to develop in the presence of normally effective doses of MLs and extends to survival of microfilariae (mf) at these doses as well [5, 6]; less has been reported about MLR phenotypes in adult stages.

Although an in vivo microfilariae suppression test is available to diagnose MLR [5, 6], no in vitro test has been deployed on the basis of ML effects on more readily accessible mf or L3 larval stages. The in vivo test is not suitable for routine use, as it requires quantitative counts of bloodstream mf and two visits to a veterinary clinic 3–4 weeks apart after initial treatment with a ML at a normally highly effective dose; MLR is suggested if the dose fails to reduce microfilaremia by > 90% [6]. Genome sequencing efforts identified multiple single nucleotide position (SNP) markers that robustly distinguish MLR from wt heartworms on the basis of in vivo phenotype-genotype correlation [6]; further work showed that a combination of two of these markers was highly correlated with MLR [7–9]. Polymerase chain reaction (PCR)-based tests based on these two SNP markers were used to map the epidemiology of MLR heartworms in dogs in the USA [10, 11]. In one, analysis of mf isolated from clinical samples in southwest Missouri revealed that 95% were positive for the MLR markers [10]. A second study reported that approximately one-third of 310 mf samples obtained from clinics across the USA could be classified as MLR on the basis of the SNP markers and another one-third could be classified as mixed resistant and susceptible [11]. These cases all reflect dogs that were patently infected with *D. immitis* and cannot be used to directly estimate the overall prevalence of MLR versus susceptible parasites in the geographic area. In addition, the treatment history of the sampled dogs was not available. Nonetheless, these recent results are a cause for concern.

A possible limitation of these kinds of studies is that the diagnostic SNPs for MLR do not lie in a gene that can be considered causative for the phenotype; confirmation that these parasites are phenotypically resistant should be pursued. It is very unfortunate that the molecular basis for MLR in heartworms remains unknown. Despite this, additional surveys for the presence and prevalence of the available MLR markers in mf from infected dogs should be a priority.

Therapeutic consequences of MLR heartworms remain under investigation. Studies have shown that 4–6 monthly doses of moxidectin-containing products and an extended release parenteral moxidectin product retain very high, though not quite 100%, efficacy against two AR isolates of the parasite [12–16], while preventative products containing ivermectin or milbemycin oxime were significantly less effective in the same regimen [13, 14]. Single doses of any ML preventative product fall short of 100% efficacy against known MLR isolates; data on the consequences for prevention of a patent infection if one or more doses in a monthly series is missed have not been reported.

The pharmacological bases for the greater efficacy at an approved dose of moxidectin compared with other MLs are suggested to include the higher intrinsic potency and longer half-life of moxidectin [see 17]. Of note, the cumulative duration of exposure to an ML preventative as a variable for efficacy against MLR parasites has not been sufficiently investigated.

## Knowledge gaps and research priorities

It is challenging to obtain funding to study MLR in heartworms at an academic level, a situation made more difficult by the requirement to work with dogs as experimental animals and to maintain an insectary capable of housing vector mosquito species to supply the life cycle of the parasite. Notwithstanding these challenges, research into MLR is urgently needed to be able to provide sustainable prevention of heartworm infections into the future. Several priority research areas, in no order of urgency, are outlined below.

### More well-characterized heartworm isolates are needed and should be shared

Most studies reported have employed only one or a few MLR isolates of *D. immitis*. Relatively little is known about how these isolates have been maintained in host canines, including information on the total number of passages and the number made in the presence of an ML. Since these isolates are not clonal, each sampling for a passage will select a variable number of WT and MLR individuals unless mf are obtained only from dogs treated with a normally effective dose of an ML. Although fitness deficits are not evident in MLR populations in mosquitoes [18], we do not know whether this is the case in the canine stage of the lifecycle. Larval stages of *D. immitis*, including mf and L3, could be cryopreserved [e.g., 19] to maintain a resource of well-characterized isolates for experimental use if an appropriate reference laboratory can be established to house them. Standardization of naming conventions for isolates would be facilitated by a common source of parasites such as this [20].

### Alternative heartworm models are needed

Several immunocompromised rodent models have been developed for heartworm research [21–24]. None of these hosts produce enough mf to allow the life cycle to be maintained without dogs, but these nonetheless provide valuable alternatives to enable better characterization of MLR. Notably, a model using a common rat strain immunocompromised by steroid feeding patented by Zoetis detects ML sensitivity at regimens comparable to those used clinically in dogs [24] and has many potential advantages, including much lower expense of animals and housing compared with genetically immunosuppressed mouse strains. The rat model could be used, if permission can be obtained for noncommercial research purposes, to characterize differences among MLs (shift to the right). For example, the quantitative extent to the shift to the right of the dose–response curve could be measured to determine whether individual MLs vary in this characteristic and whether different isolates of resistance heartworms show the same extent of resistance. Duration of exposure as a variable for efficacy could be measured by including daily or weekly treatments with MLs compared with sequential monthly regimens.

### Culture-based phenotypic assays are needed

Culture-based phenotypic assays using larval stages of gastrointestinal nematodes (GIN) have proven useful in identifying AR isolates even in the absence of knowledge of molecular mechanisms of resistance, including a variety of developmental and motility parameters such as egg hatch, larval development, and larval migration assays [25]. Unlike in GIN, the antifilarial action of MLs does not appear to involve inhibition of motility of larval stages [see 26]. Instead, evidence suggests that these drugs block the release of immunomodulatory factors from the parasite, allowing the canine immune system to recognize and attack them [26–31]. The potency of MLs for inhibition of secretion reflects the resistance status of the heartworm isolate [30, 31], suggesting a possible strategic path for developing an in vitro diagnostic assay for the phenotype.

Attempts have been made to use protein secretion from mf in culture as an assay for MLR by measuring activity of abundantly secreted enzymes [e.g., 32], but interpretation of results vis-à-vis resistance status proved challenging. Additional research, perhaps using immunoassay platforms to measure protein release from mf exposed to MLs, might provide a useful way to quantify ML potency in larvae in culture. Without a commercially available molecular test for MLR, it will be difficult to monitor the extent and spread of these parasites. A culture-based assay that could be conducted in a research laboratory would be highly beneficial until a commercial test is developed.

### Pharmacological unknowns

As noted, we do not know the quantitative extent of MLR, or whether all MLs and all isolates are equal in this regard. As MLR developed in other species of parasitic nematodes, continued use of these anthelmintics tended to increase the degree of resistance. Although phenotypic resistance to moxidectin was less severe or even absent initially, its continued use eventually led to resistance to it as well. We do not know whether extended courses of oral moxidectin or continuous exposure via parenteral products will eventually lead to phenotypic resistance beyond the current extent, especially if moxidectin-containing products become dominant in practice. Given the expense and ethical difficulties of conducting drug challenge experiments in heartworm-infected dogs, it is almost certain that the answer to the question of whether moxidectin-resistant isolates will evolve will only come from clinical observations.

Although not approved for this use, it must be recognized that moxidectin-containing products are also used in slow-kill regimens for dogs infected with adult stages of heartworms [e.g., 33, 34]. The merits of this approach compared with standard melarsomine-doxycycline regimens are not the subject of this discussion; rather, the issue is whether MLR status influences the ability of moxidectin to resolve adult stage infections. As is the case for many priorities for research into MLR in *D. immitis*, it is unlikely that a systematic evaluation of this issue will be carried out. Instead, dogs that receive slow kill treatment for adult heartworms should be monitored regularly for evidence of mf reduction and conversion to adult antigen negative status. Clinical failure with a slow kill regimen may require arsenical treatment to remove the adult worms.

### The causative gene(s)/mechanism of resistance of ML resistance

Whole genomic sequences of varying quality have been reported for many isolates of *D. immitis* [4, 35–40]. Comparisons of genome sequences of ML-susceptible versus ML-resistant isolates revealed multiple distinguishing SNP alleles [4] but have not led to identification of a gene or genes that cause the resistant phenotype.

The situation is similar to what has been found in other species of parasitic nematodes. Studies of *Haemonchus contortus* in sheep involving crosses of strains of ML-susceptible and ML-resistant parasite strains, followed by whole-genome analysis, pinpointed a region on chromosome 5 as the most likely source of the phenotype [41]. The authors identified the transcription factor *cky-1* as the most likely causative gene underlying ML resistance in this parasite. Further work suggested that this transcription factor is overexpressed in pharyngeal tissue in MLR versus MLR-susceptible strains of *H. contortus* [42], but the critically important downstream genes regulated by this transcription factor were not identified. Whether changes in expression of this factor occur in MLR isolates of other parasitic nematodes, including *D. immitis*, is not clear.

Available isolates of MLR *D. immitis* are not clones, and depending on the history of passage through mosquitoes and dogs in the presence of ML treatment, may consist of a mix of susceptible and resistant individuals; if resistance is a polygenic trait in this parasite, individuals with a variable extent of resistance are also likely to be present. The ability to generate more highly inbred strains of heartworms could facilitate the identification of the gene(s) underlying MLR but await investment in the expensive and time-consuming experiments (currently requiring dogs) needed to generate them.

## Multiple anthelmintic drug resistant (MADR) canine hookworms: implications for heartworms

The canine hookworm *Ancylostoma caninum* is widely distributed in dogs in North America [see 43]. Populations of this parasite resistant to all anthelmintic classes have been selected and are now widely spread across the continental USA and have been found in Canada. These parasites do not directly affect the pathology, epidemiology, or treatment of heartworm infections, but a complication relevant for heartworm therapy has arisen from the extra-label use of emodepside, a cyclooctadepsipeptide anthelmintic, to treat unresolvable MADR *A. caninum* cases [43]. Emodepside is an active ingredient in Profender^®^ (Elanco) products that are registered for use in cats as a topical product in the USA; although Profender^®^ (Vetoquinol) products are registered for use in dogs for oral administration in many countries, they are not approved in the USA. Emodepside is highly effective in controlling gastrointestinal parasitic nematodes in cats and dogs, including MADR hookworms [see 43]. Treatment of MADR hookworms in the USA typically involves oral administration of the cat topical product, which results in threefold higher Cmax values than the oral product licensed in other countries [44], adding to the safety concerns.

It must be recognized that the extra-label use of emodepside in MADR hookworm cases poses serious risks for dogs co-infected with heartworms. Emodepside is a highly active filaricide, lethal to all stages of many species of filariae in many host species [45]. Indeed, it is in clinical development for human use as a macrofilaricide for onchocerciasis and for human GIN infections [46, 47]. The filaricidal activity of emodepside extends to *D. immitis*; it is quite potent (3 nM) in culture against adult and larval stages of heartworms [46]. There is thus a risk of adverse events due to the killing of adult stages (or L5s) in heartworm-infected dogs treated with emodepside for MADR hookworm infections. Dogs should be tested for heartworm infection before being treated with emodepside and clients should be fully informed about potential risks, as would be the case for standard adulticidal therapy. It is also important to recognize that emodepside is a substrate for the MDR-1 P-glycoprotein that protects the CNS from drugs, and that dogs with loss-of-function mutations in the gene encoding this transporter may be at increased risk of adverse events if treated with the drug [48, 49], especially considering the pharmacokinetic profile associated with oral administration of the feline topical product.

## Implications for AR in companion animal parasites

The concept of refugia has been an extremely important contributor to understanding the selection, evolution, and spread of AR [see 50 for review]. The proportion of a parasite population that remains untreated with anthelmintics and the extent to which treated and untreated parasites interbreed determines in part the rate at which AR can develop. Nematode parasites of companion animals have been thought to be examples for which refugia is extensive, delaying the development of AR, because a large percentage of companion animals do not receive anthelmintic treatment and wildlife hosts represent another pool of untreated pathogens. However, companion animal parasites have developed AR [51], and the spread of AR populations of heartworms and canine hookworms requires an adjustment in our understanding of refugia. In both situations, AR is thought to have arisen in locations in which intensive parasite transmission was addressed with intensive anthelmintic treatment and in which introduction of new (untreated) parasites was minimal.

For heartworms, kennels in the lower Mississippi River system are thought to have been the initial selection site of MLR parasites. Intensive transmission occurs all year in this area and kennels may hold relatively constant canine populations with limited introduction of new animals infected with untreated wt parasites. Of critical importance in this regard is the spatial range of local vector mosquito species, some of which tend to remain quite close to the site of oviposition and thus more likely to transmit only parasites present in local, intensively treated dogs. This scenario would create pockets of highly limited refugia in an endemic area, facilitating the selection of resistant parasites.

Similarly, MADR hookworm populations are thought to have arisen in greyhound racing operations in the southern USA (Florida) and perhaps in other kennels elsewhere [see 43]. These operations experienced conditions favorable for intensive transmission as well as development of infective larvae of hookworms and treatment with all classes of anthelmintics registered for use in dogs in the USA to maximize performance, with limited introduction of wt parasites, fostering selection of MADR parasites. Adoption of greyhounds from the racing operations led to dispersal of MADR hookworms across the continental USA and into Canada [see 43].

Veterinarians and veterinary parasitologists should pay close attention to operations in which dogs are housed under conditions in which heartworm transmission is intensive and heartworm prevention is rigorously practiced, particularly if new dogs bearing untreated parasites are rarely introduced. Although mosquito surveys may not be commonly done, it would be beneficial to identify areas in which intensive preventative treatment is practiced and in which vector mosquitoes remain close to hatching sites, limiting the introduction of untreated parasites via vectors. Such areas should receive added attention for the possible presence of MLR heartworm infections.

## Conclusions

Anthelmintic resistance has developed and spread in two species of nematodes that parasitize dogs in North America [51]. Populations of MLR heartworms have been selected, and on the basis of recent epidemiological studies using molecular markers linked to this trait, have spread in the continental USA beyond the lower Mississippi River valley where they first appeared [10, 11]. Rigorous compliance with monthly use of high-dose moxidectin-containing products still achieves almost complete protection against known MLR isolates of *D. immitis* in laboratory settings [12–16], but whether all circulating parasite populations are the same remains to be determined, and whether continued use of these products will eventually select isolates with greater resistance is unknown. Veterinarians and veterinary parasitologists should be vigilant in monitoring efficacy of heartworm preventatives, particularly in situations of low local refugia. More research is needed to identify the genetic cause of MLR and to develop assays that can be used to monitor it.

Canine hookworms (*A. caninum*) have developed MADR, posing significant challenges for treatment and control [43]. Extra-label emodepside has appeared in an attempt to resolve otherwise recalcitrant hookworm infections, using oral administration of the cat topical product. While highly effective against *A. caninum*, emodepside is a potent macrofilaricide and may cause adverse reactions if given to dogs bearing immature or mature adult *D. immitis* and in dogs with null MDR-1 mutations [43]. Studies to determine the safety of emodepside in patent heartworm infections are unlikely to be conducted, and practitioners should use caution in such cases, with fully informed consent.

## Data Availability

Data supporting the main conclusions of this study are included in the manuscript.

## Abbreviations

USA: United States of America
ML: Macrocyclic lactone
MLR: Macrocyclic lactone resistant/ce
MADR: Multiple anthelmintic drug resistant
AR: Anthelmintic resistance
WT: Wild type
mf: Microfilariae
SNP: Single nucleotide polymorphism
PCR: Polymerase chain reaction
GIN: Gastrointestinal nematode
